# Cost-effectiveness of a nurse-led telemonitoring intervention based on peak expiratory flow measurements in asthmatics: results of a randomised controlled trial

**DOI:** 10.1186/1478-7547-5-10

**Published:** 2007-07-27

**Authors:** Daniëlle CM Willems, Manuela A Joore, Johannes JE Hendriks, Emiel FM Wouters, Johan L Severens

**Affiliations:** 1 Department of Clinical Epidemiology and Medical Technology Assessment, University Hospital Maastricht, PO Box 5800, 6202 AZ Maastricht, The Netherlands; 2 Department of Paediatrics, University Hospital Maastricht, PO Box 5800, 6202 AZ Maastricht, The Netherlands; 3 Department of Respiratory Medicine, University Hospital Maastricht, PO Box 5800, 6202 AZ Maastricht, The Netherlands; 4 Department of Health, organisation, policy and Economics, Maastricht University, PO Box 616, 6200 MD Maastricht, The Netherlands

## Abstract

**Background:**

Asthma is a chronic lung disease in which recurrent asthma symptoms create a substantial burden to individuals and their families. At the same time the economic burden associated with asthma is considerable.

**Methods:**

The cost-effectiveness study was part of a single centre prospective randomised controlled trial comparing a nurse-led telemonitoring programme to usual care in a population of asthmatic outpatients. The study included 109 asthmatic outpatients (56 children; 53 adults). The duration of follow-up was 12 months, and measurements were performed at baseline, 4, 8, and 12 months. Patients were asked to transfer their monitor data at least twice daily and by judging the received data and following a stepwise intervention protocol a nurse was able to act as the main caregiver in the intervention group. In both groups the EQ-5D and the SF-6D were used to obtain estimates of health state utilities. One year health care costs, patient and family costs, and productivity losses were calculated. The mean incremental costs were weighted against the mean incremental effect in terms of QALY.

**Results:**

The study population generally represented mild to moderate asthmatics. No significant differences were found between the groups with regard to the generic quality of life. Overall, the mean health care costs per patient were higher in the intervention group than in the control group. The intervention costs mainly caused the cost difference between the groups. The intervention costs the society € 31,035/QALY gained with regard to adults and with regard to children € 59,071/QALY gained.

**Conclusion:**

If the outcome is measured by generic quality of life the nurse-led telemonitoring programme is of limited cost-effectiveness in the study population. From the societal perspective the probability of the programme being cost-effective compared to regular care was 85% at a ceiling ratio of € 80,000/QALY gained among the adults and 68% among the children. A decrease in the price of the asthma monitor will substantial increase the probability of the programme to be cost-effective.

**Trial registration:**

Number: NCT00411436

## Background

Asthma is a chronic lung disease from which worldwide 300 million people suffer. Among children it is even the most common chronic disease. Recurrent asthma symptoms create a substantial burden to individuals and their families and lead to restriction of an individual's activities. Asthma can be successfully controlled with proper care, which enables patients to enjoy good quality of life [1]. In a continuous cycle therapy should be adjusted by assessment, treatment, and monitoring, depending on the patient's level of control [2]. If asthma is not in control it can lead to increase in medical consumption and to school and work absenteeism. Consequently the economic burden associated with asthma is considerable [1]. In the year 2000 the total costs for an asthma patient were on average € 312 in the Netherlands. Medication costs were the largest cost component in the direct costs (53%). Furthermore, the medication costs were estimated to triplicate by the year 2025 [3]. A study performed in 2003 in the USA has concluded that caring for a severe asthmatic costs $12,813 a year (approximately € 10,000) [4]. The largest components in the direct costs were medication (53%) and hospital admissions (15%). Since financial resources are scarce and technology is improving economic evaluations are helpful in making decisions among different health care interventions. This is particularly useful to decide if a new health care technology should be implemented. Economic evaluations present a comparative analysis of alternative courses of actions in terms of both their costs and consequences. The incremental cost effectiveness ratio is defined as the difference in costs between two technologies divided by the difference in their benefits. The lower the incremental cost effectiveness ratio, the more cost effective a technology [5].

Telemonitoring is a recent development in healthcare and offers new method to manage asthma. By the use of telemonitors, spirometry tests can continuously be monitored by transferring monitor data from the patient's home to a central database. This monitoring at a distance is very useful in the management of asthma, since asthma cannot be cured and the presence of asthma symptoms can change every day. In a recent literature review it was stated that long-term disease monitoring of patients at home stimulates cost-effectiveness in health care [6]. Nurses increasingly play a part in telemonitoring and nurse-led telemonitoring programmes in chronic diseases are commonly considered cost-effective. By employing a nurse (practitioner) as the main caregiver costs can be decreased to a minimum, while the quality of care stays intact [7]. However, only a few studies have calculated costs or incremental costs-effectiveness ratios [8]. To our knowledge no study has performed a cost-effectiveness analysis of a nurse-led telemonitoring intervention in asthmatic outpatients.

The objective of this study was to determine the cost-effectiveness of a nurse-led telemonitoring programme in asthmatic outpatients compared to regular care. The study protocol was approved by the appropriate ethics committee and informed consent was obtained from each participant.

## Methods

### Data source

The cost-effectiveness study was part of a single centre prospective randomised controlled trial comparing a nurse-led telemonitoring programme to usual care in a population of asthmatic outpatients. Randomisation took place on patient level after stratification by age (children aged 7 to 18 versus adults aged 18 years and older). The duration of follow-up was 12 months, and measurements were performed at baseline, 4, 8, and 12 months.

The study population consisted of asthmatic outpatients from the Medical Respiratory Department and the Department of Paediatrics at the University Hospital Maastricht in the Netherlands. Patients aged 7 and older with an asthma severity of stage I – III as described in the Gina guidelines were potentially eligible. The patients had to be competent to use an asthma monitor, and had to possess a household phone connection. Exclusion criteria were severe co-morbidity (such as cystic fibrosis or congenital lung abnormalities), since these patients frequently require outpatient visits. The inclusion and exclusion criteria were verified by the use of patient records. The power calculation for this study was based on improvement in asthma-specific quality of life, with a minimal important difference of 0.5 points on the 7-point overall scale in the Asthma Quality of Life Questionnaire (AQLQ) or the Paediatric Asthma Quality of Life Questionnaire (PAQLQ) [9-11]. One-sided testing, a power of 80%, and a significance level of 0.05 with a drop out percentage of 10% resulted in a minimal inclusion of 51 patients in the intervention group and 51 patients in the control group.

This study focuses on the cost-effectiveness analyses. The process evaluation and the effects of the main outcome parameters ((P)AQLQ) are described elsewhere [12].

### Comparators

The control group received regular outpatient care. In case of stable asthma these patients received three to six monthly medical check ups by their lung specialist or paediatrician. In case of exacerbations the patients received additional care from their general practitioner and/or outpatient care. The intervention group used an asthma monitor at home, and had a hospital-based nurse practitioner (also referred to as the asthma nurse) as the main caregiver. The monitor was a portable hand-held device with a matching modem. Patients were able to review their spirometry test results on the monitor screen. Transfers of the monitor data to the nurse practitioner's computer were performed by the patients by connecting the modem to the household phone. Patients were instructed to perform daily lung function tests (both in the morning and in the evening) and more often if they were having symptoms. Patients were asked to transfer the monitor data to the nurse practitioner every month and more frequently if they were having asthma symptoms. Patients with less serious symptoms were instructed to call the nurse practitioner during their programme participation. The nurse practitioner studied the data and classified the asthma following a stepwise intervention protocol. According to this protocol the nurse practitioner was allowed to decrease (after 3 months of stable asthma) or increase (if asthma was unstable) asthma medication by one step.

In case of an exacerbation the nurse practitioner judged if the exacerbation was only an incident or more structural. If he concluded that this was structural the nurse practitioner had to increase the treatment by one therapy step as described in the protocol. If standard treatment of an exacerbation was not successful the nurse practitioner contacted the treating physician to decide whether or not a steroid course was necessary. In addition, according to the protocol every 3 months the medication could be changed (1 step up or down) by the nurse practitioner. If the PEF curve was within normal range and the symptoms were minimal the nurse practitioner asked the patients to decrease the treatment level by one step. A physician was only consulted if necessary. Following this procedure, the nurse practitioner could adjust or maintain the treatment due to the continuously monitoring.

### Effects

Two multi-attribute utility instruments were used to obtain estimates of health state utilities: the EQ-5D and the SF-6D. Both instruments were administered at baseline, and at 4, 8, and 12 months follow-up.

The EQ-5D consists of a descriptive system and a visual analogue scale [13]. The five questions in the EQ-5D classify persons into one of 243 health states. The commonly used scoring function for the EQ-5D is based on a British study (EQ-5D UK) with preferences derived by the time trade-off-method (TTO), in a representative sample of the UK population [14]. The possible range of utility scores is from -0.59 to 1.00. In the subgroup of children the EQ-5D child version as previously used by Stolk et al. [15] was used. Children aged 12 years and older completed this version themselves, for children younger than 12 years of age the parents or caregivers completed the child proxy version.

The SF-6D is derived from the SF-36 [16]. The SF-6D instrument covers 6 domains, and describes 18,000 health states. Using a fractional factorial design, 249 health states were identified and valued by a representative sample of the UK general population using the standard gamble valuation method [17]. An algorithm provided by Brazier and colleagues was used to construct the utilities. The utility scores range from 0.29 to 1.0. SF-6D utilities could only be obtained in the subgroup of adult participants because no child version is available.

### Costs

Costs can be divided into health care costs, patient and family costs, productivity losses, and costs in other sectors [5]. Health care costs included costs associated with hospital care, general practitioners and other health care professionals, prescribed medication, professional home care, and the intervention. Hospital care included day admissions, emergency room visits, surgical and diagnostic procedures, laboratory research, and outpatient visits. Patient and family costs consisted of the costs of over-the-counter medication, and informal care. Productivity losses include the costs due to productivity loss at paid and unpaid work. Costs in other sectors are the resources consumed in other sectors, such as volunteer work and nursing home care. These costs were absent in this study and therefore not included in the analysis. The volumes of hospital care were obtained from the hospital billing system of the university hospital Maastricht. All other resource use was collected using a four weeks prospective cost diary completed at 1, 4, 8 and 12 months follow-up. Cost diaries have proven to be a successful means to gather information on healthcare resource use during a longer period [18]. The data from each cost diary were interpolated (multiplied by 3) in order to obtain estimates of resource use during the entire one year follow-up period.

Unit prices from the Dutch manual for cost research were used if available [19]. Costs associated with the loss of productivity at volunteer work or household activities were calculated using a shadow price of € 8.30 per hour of absence. Costs from productivity loss at paid work were calculated according to the friction cost method [20]. This method calculates productivity loss costs for the duration of the friction period. The friction period is the theoretical time needed to fill a vacancy as a result of illness. This method is recommended by the Dutch guidelines for pharmaco-economic research [21]. Costs of school absenteeism were incorporated in a sensitivity analysis. This calculation was mainly based on data of the Ministry of Education, Culture and Science [22]. The hours of school absenteeism were derived from the cost diaries. The costs associated with school absenteeism consisted of government costs and (voluntary) parental contribution and these costs depend on school type and class. The total costs were calculated by multiplying the hours of school absenteeism with the corresponding unit prices.

For the costs of the intervention a micro costing calculation was performed. The intervention costs consisted of costs of materials (the asthma monitor, and computer equipment), costs of personnel (the nurse practitioner), telephone costs, and travel costs. The price of the asthma monitor was € 476, and the price of the modem € 1428. Depreciation over five years, with 4,5 % interest, leads to annual costs of € 434 per patient. The annual costs of an insurance for the equipment amounted to € 16 per patient. The costs of the computer equipment the nurse practitioner used to receive and analyse the transferred data (a personal computer, software, monitor, and printer) amounted to € 1,150. For 55 patients, depreciated over five years, with 4,5 % interest, these costs equal € 5 per patient per year. Other fixed costs were associated with the development and production of instruction material (€ 4 and € 7 per patient per year), and administrative tasks of the nurse practitioner (€ 7 per patient per year). A continuous time registration of the activities (reviewing the lung function data the patients send in, adjusting the treatment plans, telephone contacts and house calls) of the nurse practitioner was performed. Based on the salary costs of a nurse practitioner (€ 44,700 per year), and 1540 workable hours per year, the costs of the nurse practitioner were estimated to amount to € 29 per hour. Costs of repair of the asthma monitor and modem were registered by the nurse practitioner.

Overhead costs were calculated over all direct material and personnel costs (35%) [19]. All costs were calculated for a period of one year, therefore discounting was not indicated. Costs are presented in euro for the year 2002.

### Statistical analyses

Analyses were performed by the intention-to-treat principle. Data imputations for missing values were carried out consecutively in three steps. Firstly, overall mean scores at baseline substituted missing baseline scores. Secondly, missing scores between two valid scores measured in time were individually interpolated. Thirdly, remaining missing values were imputed by Last Value Carried Forward procedure. If data was normally distributed analysis on the resulting complete data was done by repeated measures ANCOVA using 'time' as a fourth category within-patients factor and both the experimental factor (control/intervention) as well as type of patient (child/adult) as dichotomous between factors. The full model ANCOVA allowed for testing of first and second order interactions between the covariate (baseline scale scores) and both factors.

Adjustments for baseline differences in health state utility for follow-up took place. In the intervention group, for children and adults apart, the utility scores during the follow-up measurements were corrected with the mean difference in baseline utility between the intervention group and the control group. All significant test results involved 2-tailed probabilities with alpha set at 0.05. Analyses were performed according to the intention-to-treat principle. Statistical analysis was performed using SPSS, version 12.0.

### Cost-effectiveness analysis

The time horizon of the cost-effectiveness analyses was 1 year. The cost-effectiveness analysis was conducted from both the health care and the societal perspective. Adults and children were analysed separately. In the analyses from both perspectives the mean incremental health care costs were weighted against the mean incremental effect in terms of quality adjusted life years (QALY). Since no mortality occurred the QALY was calculated for each patient by multiplying each of the four measured utility values with 4 months. This calculation was performed by the use of the EQ-5D utility (adults and children) and the SF-6D utility (adults only). To obtain the incremental QALY multiple regression analysis was applied to control for the differences in baseline utility. To get insight into the uncertainty around the incremental cost-effectiveness ratio (ICER) nonparametric bootstrap simulations were conducted [23]. Bootstrapping was performed on both the incremental regression based QALY (based on both the EQ-5D utility and the SF-6D utility) versus both the incremental health care costs and the incremental societal costs. In the bootstrap simulation 5000 random samples of cost-effect pairs, of equal size of the original sample, were selected with replacement. The scatter plots represent points of which each signifies the incremental cost-effectiveness ratio of one iteration of the bootstrap simulation. From a decision-makers point of view, the probability that a new treatment is cost-effective varies depending on what society is prepared to pay per gain in effectiveness, the so-called ceiling ratio. In the Netherlands € 80,000/QALY gained has been mentioned [24]. Another mentioned ceiling ratio is € 40,000/QALY gained. This is shown in cost-effectiveness acceptability curves.

### Sensitivity analyses

One-way sensitivity analyses are performed to test for the impact of two cost components on the study results. In the first analysis the total asthma monitor costs including monitor, modem, batteries, and insurance (€ 450) were set to zero in the intervention group. In the second analysis the costs resulting from school absenteeism by children were included in the societal costs.

## Results

### Study population

From patient records of the departments of Respiratory Medicine and Paediatrics of our hospital 274 potentially eligible asthmatic outpatients were identified and approached by letter. Eighteen patients were not eligible because of the absence of a house phone connection (7%), and 147 patients refused to participate in the study (54%). The most frequently reasons of the 40 adults and 26 children who registered their reason for not participation were: 'having no time' (29% adults; 14% children), 'being uninterested' (9% adults; 11% children), 'not experiencing asthma symptoms' (5% adults; 8% children), and 'finding participation too confronting' (5% adults; 2% children). Finally 40% was included in the study. Between January 2003 and January 2004, 109 patients (53 adults and 56 children) were enrolled in the study. The characteristics of the intervention and control group appeared to be similar at baseline, except for baseline utility. The patient characteristics of the study population are presented in table 1. Of the 109 participants, seven patients (5 intervention group; 2 control group) were lost to follow-up. In total five patients of the 55 patients in the intervention group were lost to follow-up (two adults and three children). In the control group two of the 54 patients were lost to follow-up (one adult and one child). The reason to stop participation was for one patient in the control group immigration, further all lost to follow-up occurred because the patients refused further participation. Reasons for these refusals were not given. In the intervention group four cases of loss to follow-up took place immediately after the baseline measurement, and one case occurred after the third measurement at eight months.

**Table 1:** Baseline characteristics of the patients by age and group

Characteristics	Adults (18 years and older)				Children (7 – 18 years)			
	Control N = 27		Intervention N = 26		Control N = 27		Intervention N = 29	

mean (sd)								
age	45.90	(15.9)	45.65	(11.3)	10.85	(2.3)	10.57	(2.1)
lung function values								
- FVC % pred	102.5	(15.3)	104.2	(14.7)	98.7	(17.7)	96.5	(13.7)
- FEV1 % pred	92.4	(19.9)	92.6	(21.4)	99.4	(11.3)	96.5	(8.4)
- FEV1 % VC ref	75.2	(10.4)	73.0	(12.8)	84.1	(9.8)	82.4	(8.9)
- PEF % pred	99.3	(23.8)	108.4	(42.9)	91.7	(14.9)	91.3	(16.0)
GINA classification	2.74	(0.7)	2.96	(0.5)	2.07	(0.7)	2.31	(0.8)
Gender								
- male	33.3	%	42.3	%	55.6	%	72.4	%
- female	66.7	%	57.7	%	44.4	%	27.6	%
civil status								
- single	22.2	%	7.7	%	0	%	0	%
- with parents	7.4	%	0	%	100	%	100	%
- married/living together	77.4	%	92.3	%	0	%	0	%
main daily activity								
- paid employment	44.4	%	65.4	%	0	%	0	%
- sick leave/disabled	25.9	%	11.5	%	0	%	0	%
- school/college	3.7	%	0.0	%	100	%	100	%
- housekeeping	11.1	%	7.7	%	0	%	0	%
- other	14.8	%	15.4	%	0	%	0	%

FVC % pred = forced vital capacity expressed as a percentage of predicted; FEV1 % pred = forced expiratory volume in 1 second expressed as a percentage of predicted; FEV1 % VC ref = forced expiratory volume expressed as a percentage of predicted/vital capacity; PEF % pred = peak expiratory flow expressed as a percentage of predicted. GINA classification based on prescribed medication; 1 = intermittent asthma, 2 = persistently mild asthma, 3 = persistently moderate asthma, 4 = persistently severe asthma.

In the control group one patient was lost to follow-up after the baseline measurement and one patient after the third measurement at eight months.

### Effects

Generic quality of life as measured with the EQ-5D descriptive part showed little to no problems at all measurements. The scores on the VAS indicated a moderate to good self perceived health status in both groups. The population utility scores were higher than the scores on the VAS. No differences between groups in time in the VAS scores were observed in the adults (*P *= .638; ANCOVA) or the children (*P *= .521; ANCOVA). See table 2 and 3.

**Table 2:** a. EQ-5D mean (SD) domain scores and utility score for Adults (18 years and older) in Control group (N = 27) and Intervention group (N = 26; total N = 53)

EuroQol	Baseline		Month 4		Month 8		Month 12			
	Control	Intervention	Control	Intervention	Control	Intervention	Control	Intervention	*time*^1^	time*group^2^
									*P*	
Mobility	1.37 (0.49)	1.15 (0.37)	1.32 (0.46)	1.00 (0.00)	1.35 (0.55)	1.15 (0.37)	1.30 (0.47)	1.54 (0.37)	.000	.190^3^
Self care	1.07 (0.27)	1.00 (0.00)	1.04 (0.19)	1.04 (0.20)	1.11 (0.32)	1.04 (0.20)	1.22 (0.42)	1.04 (0.20)	.000	.213^4^
Daily activities	1.48 (0.51)	1.35 (0.49)	1.54 (0.50)	1.31 (0.47)	1.54 (0.57)	1.42 (0.58)	1.56 (0.58)	1.46 (0.51)	.004	.623^3^
Pain/discomfort	1.67 (0.56)	1.31 (0.47)	1.54 (0.57)	1.23 (0.43)	1.56 (0.58)	1.35 (0.49)	1.63 (0.63)	1.42 (0.50)	.000	.871^4^
Anxiety/depression	1.11 (0.32)	1.19 (0.40)	1.22 (0.42)	1.15 (0.37)	1.19 (0.40)	1.27 (0.45)	1.19 (0.40)	1.15 (0.37)	.001	.505^4^
VAS	67.33 (17.20)	74.57 (12.78)	71.67 (17.63)	73.08 (14.29)	68.11 (19.06)	71.35 (18.45)	72.26 (18.43)	74.50 (15.87)	.081	.638^4^
Utility	0.78 (0.17)	0.89 (0.13)	0.80 (0.18)	0.91 (0.12)	0.78 (0.24)	0.86 (0.19)	0.79 (0.21)	0.90 (0.11)	.010	.596^3^

b. EQ-5D mean (SD) domain scores and utility score for Children (7 to 18 years) in Control group (N = 27) and Intervention group (N = 29); total N = 56										

EuroQol	Baseline		Month 4		Month 8		Month 12			
	Control	Intervention	Control	Intervention	Control	Intervention	Control	Intervention	*time*^1^	time*group^2^
									*P*	

Mobility	1.00 (0.00)	1.00 (0.00)	1.00 (0.00)	1.00 (0.00)	1.00 (0.00)	1.00 (0.00)	1.00 (0.00)	1.04 (0.19)	-	.428^4^
Self care	1.00 (0.00)	1.00 (0.00)	1.00 (0.00)	1.00 (0.00)	1.00 (0.00)	1.00 (0.00)	1.00 (0.00)	1.00 (0.00)	-	-
Daily activities	1.15 (0.36)	1.21 (0.49)	1.07 (0.27)	1.09 (0.30)	1.04 (0.19)	1.14 (0.35)	1.11 (0.32)	1.14 (0.35)	.000	.621^4^
Pain/discomfort	1.04 (0.19)	1.18 (0.47)	1.04 (0.19)	1.01 (0.03)	1.04 (0.19)	1.04 (0.19)	1.07 (0.27)	1.07 (0.26)	.000	.807^3^
Anxiety/depression	1.11 (0.32)	1.10 (0.31)	1.04 (0.19)	1.00 (0.00)	1.07 (0.27)	1.04 (0.19)	1.15 (0.36)	1.00 (0.00)	.000	.064^3^
VAS	81.36 (11.70)	79.61 (13.67)	84.32 (10.72)	81.29 (12.02)	83.32 (13.11)	82.59 (11.39)	81.33 (14.69)	82.31 (12.49)	.000	.521^4^
Utility	0.96 (0.07)	0.92 (0.20)	0.98 (0.07)	0.99 (0.03)	0.98 (0.06)	0.98 (0.07)	0.97 (0.05)	0.98 (0.04)	.000	.557^3^

P-values based on Repeated Measures ANCOVA corrected for baseline; performed separately for age group (adults/children).

^1 ^p-value for overall follow-up in time.

^2 ^p-value for follow-up difference in time between groups.

^3 ^Greenhouse-Geisser.

^4 ^Sphericity Assumed.

- = analyses could not be performed due to minimal to zero variance

Overall, the adults in the intervention group experienced fewer problems with regard to the items of the EQ-5D including the baseline measurement then the control group. In time, the domain scores all significantly improved in both groups. Between the groups in time no statistically significant differences were observed. SF-6D scores and utility were relatively high, and slightly higher in the intervention group at all measurements. The results of the SF-6D among the adult participants showed the same pattern as the scores on the EQ-5D. Differences between the groups in time were not statistically significant. See table 4.

**Table 3:** SF36 mean (SD) domain scores and SF-6D mean (SD) utility score for adult (18 years and older) study participants in the Control group (N = 27) and Intervention group (N = 26)

SF36	Baseline		Month 4		Month 8		Month 12		*time*^1^	time* group^2^
	C	I	C	I	C	I	C	I	*P*	
Physical functioning	68.06	72.42	67.05	75.62	66.02	71.20	68.79	71.58	.088	.412^4^
	(24.3)	(20.9)	(25.7)	(21.5)	(26.9)	(23.9)	(25.7)	(22.6)		
Social functioning	66.20	75.96	71.99	76.92	71.99	74.52	72.22	78.37	.000	.743^4^
	(26.4)	(26.4)	(26.3)	(20.52)	(27.0)	(25.4)	(25.6)	(22.0)		
Role physical	42.59	49.85	48.61	56.73	54.17	47.12	57.41	53.53	.001	.327^4^
	(46.4)	(44.2)	(43.9)	(43.9)	(47.2)	(45.5)	(45.9)	(45.5)		
Role emotional	65.50	71.80	65.43	71.80	76.54	67.95	77.78	78.21	.000	.438^4^
	(41.8)	(41.8)	(45.0)	(41.8)	(41.2)	(46.7)	(40.3)	(38.8)		
Mental health	71.74	75.29	74.30	71.39	71.56	73.69	76.59	77.23	.004	.335^3^
	(17.4)	(18.7)	(17.7)	(21.5)	(18.9)	(22.2)	(17.6)	(18.4)		
Vitality	52.96	58.68	53.92	59.23	53.61	55.77	59.44	60.77	.074	.574^3^
	(19.6)	(20.9)	(17.7)	(22.4)	(21.3)	(21.7)	(22.9)	(23.6)		
Bodily Pain	64.96	75.15	66.37	84.35	65.26	72.00	67.59	74.92	.000	.064^4^
	(25.6)	(24.4)	(25.2)	(18.9)	(24.21)	(24.9)	(23.3)	(21.7)		
General Health	48.55	47.31	48.83	51.72	48.65	50.89	52.05	50.05	.011	.459^3^
	(21.8)	(22.0)	(22.40)	(21.7)	(23.9)	(24.2)	(24.2)	(21.3)		
SF-6D utility	0.69	0.75	0.72	0.75	0.71	0.74	0.74	0.75	.012	.301^3^
	(0.14)	(0.13)	(0.14)	(0.13)	(0.16)	(0.15)	(0.14)	(0.14)		

C = control group; I = intervention group.

Domain scores range 0–100.

P-values based on Repeated Measures ANCOVA corrected for baseline.

^1 ^p-value for overall follow-up in time.

^2 ^p-value for follow-up difference in time between groups.

^3 ^Greenhouse-Geisser.

^4 ^Sphericity Assumed.

The children in the control group experienced more problems with regard to anxiety/depression on the EQ-5D at month 12 compared to the intervention group. The mean utility scores were slightly higher in the control group at baseline and all equalised during follow-up measurements. In both groups the domain scores statistically improved in time, but no statistical significant improvement between the groups were observed.

### Costs

Table 5 presents the mean (SD) costs for adults (18 years and older) and children (7 to 18 years) separate. The mean one year intervention costs amounted to € 530 (SD € 57) per adult and € 537 (SD € 54) per child. The largest part of the intervention costs was associated with the fixed costs (€ 473), and on average only a small proportion of the intervention costs was variable (€ 37, SD € 12). Overall, the mean health care costs per patient were higher in the intervention group (€ 2,228, SD € 1,582 adults; € 1,193, SD € 582 children) than in the control group (€ 1,720, SD € 1,742 adults; € 588, SD € 850; children). The cost difference between the groups was mainly caused by the intervention costs. Apart of the intervention costs, other costs were also slightly higher in the intervention group. Besides the intervention costs, in both groups medication costs and costs of professional home care accounted for a large proportion of the health care costs.

**Table 4:** Mean (SD) total costs per patient over one year for total group, and for Adults (18 years and older) and Children (7 to 18 years) separate

**Cost component**	Unit costs in 2002 €	Adults mean (SD) in 2002 €		Children mean (SD) in 2002 €	
		*Intervention N = 26*	*Control N = 27*	*Intervention N = 29*	*Control N = 27*

General practitioner practice					
general practitioner visit	20.20/visit^1^	*41 (69)*	*42 (69)*	*13 (24)*	*27 (106)*
general practitioner telephone visit	10.10/visit^1^	*4 (10)*	*5 (16)*	*3 (12)*	*0 (1)*
assistant visit	20.20/visit^1^	*8 (20)*	*17 (62)*	*11 (29)*	*4 (12)*
assistant telephone visit	10.10/visit^1^	*6 (19)*	*11 (31)*	*8 (17)*	*7 (15)*
nurse practitioner visit	20.20/visit^1^	*0 (0)*	*14 (59)*	*2 (11)*	*0 (1)*
Hospital care					
day admission	229.00/admission^1^	*0*	*8 (44)*	*2 (4)*	*27 (132)*
emergency room visit	139.00/visit^1^	*0*	*5 (27)*	*2 (4)*	*33 (161)*
surgical procedures	costs/procedure^2^	*54 (274)*	*0*	*4 (7)*	*12 (46)*
diagnostic procedures	costs/procedure^2^	*15 (55)*	*64 (208)*	*43 (72)*	*39 (71)*
laboratory research	costs/procedure^2^	*79 (379)*	*8 (37)*	*44 (126)*	*15 (35)*
lung specialist outpatient visit	100.00/visit^1^	*26 (81)*	*25 (80)*	*0*	*0*
paediatric lung specialist outpatient visit	100.00/visit^1^	*0*	*0*	*57 (180)*	*128 (358)*
asthma nurse practitioner outpatient visit	62.72/visit^3^	*104 (287)*	*17 (51)*	*55 (131)*	*17 (72)*
other medical specialists outpatient visit	100.00/visit^1^	*62 (193)*	*139 (265)*	*69 (146)*	*6 (21)*
Other healthcare professionals					
speech therapist	25.00/visit^1^	*0*	*8 (43)*	*0*	*0*
homoeopath	52.50/visit^4^	*18 (68)*	*0 (1)*	*33 (129)*	*1 (2)*
company medical officer	51.61/visit^5^	*7 (30)*	*6 (29)*	*0*	*0*
Prescribed medication					
medication	drug costs^6^	*670 (353)*	*655 (763)*	*236(241)*	*199 (247)*
pharmacist fee	6.45/prescription^1^	*60 (30)*	*59 (33)*	*37 (22)*	*40 (22)*
Professional home care	26.70/hour^1^	*548 (1327)*	*634(1170)*	*35 (117)*	*33 (108)*
Intervention costs	cost calculation^7^	*530 (57)*	*-*	*537 (54)*	*-*

**Subtotal health care costs**		*2,228 (1,582)*	*1,720 (1,742)*	*1,193 (582)*	*588 (850)*

Over the counter medication	out-of-pocket costs^7^	*7 (18)*	*5 (18)*	*4 (10)*	*0 (1)*
Informal care	8.30/hour^1^	*127 (323)*	*62 (214)*	*3 (12)*	*3 (11)*

**Subtotal patient and family costs**		*2,361 (1,673)*	*1,787 (1,794)*	*1,200 (591)*	*592 (855)*

Loss of productivity at volunteer work	8.30/hour^1^	*20 (61)*	*12 (57)*	*1 (3)*	*1 (3)*
Loss of productivity at household work	8.30/hour^1^	*105 (264)*	*50 (150)*	*5 (17)*	*5 (15)*
Loss of productivity at paid labour	friction costs^3,7^	*487 (1394)*	*99 (281)*	*0*	*0*

**Subtotal productivity losses**		*612 (1,390)*	*161 (352)*	*6 (20)*	*6 (18)*

**Total costs**		*2,973 (2,650)*	*1,948 (1,777)*	*1,206 (601)*	*597 (863)*

Notes: ^1 ^Oostenbrink, 2004. ^2 ^Hospital billing system. ^3 ^calculated from Oostenbrink et al. ^4 ^Dutch Association of Classic Homoeopaths, NVKH. ^5 ^Occupational health Service. ^6 ^Dutch Health Insurance council; . ^7 ^cost diary.

With regard to the adults the costs of informal care were on average twice as high in the intervention group (€ 127, SD € 323), as in the control group (€ 62, SD € 214). As a result the difference in patient and family costs between the groups is somewhat larger than the difference in health care costs. The costs due to loss of productivity at paid labour are on average five times higher among the patients in the intervention group (€ 487, SD € 1,394), than among the patients in the control group (€ 99, SD € 281). As a result, the total costs for adults and children are higher in the intervention group (€ 2,973, SD € 2,650 adults; € 1,206, SD € 601 children), than in the control group (€ 1,948, SD € 1,777 adults; € 597, SD € 863 children).

### Incremental Cost-effectiveness

The results of the incremental analysis are listed in table 5. Among the adults the health care costs were on average € 508 higher in the intervention group. Based on the bootstrap simulation, the 95% confidence interval (CI) of the difference in health care costs ranged from -€ 114 to € 1,118. The difference in societal costs was larger (€ 1,026; 2.5^th ^percentile € 231; 97^th ^percentile € 1,889), due to the higher productivity losses in the intervention group. Based on EQ-5D utility, after adjustment for baseline differences by multiple regression, on average 0.03 QALY (2.5^th ^percentile 0.00; 97^th ^percentile 0.07 bootstrap analysis) was gained from the intervention. The mean incremental cost-effectiveness ratio was € 15,366/QALY gained from the health care perspective and € 31,035/QALY gained from the societal perspective. The cost-effectiveness acceptability curve shows that the probability of cost-effectiveness was 85% at a ceiling ratio of € 80,000/QALY gained and 59% at a ceiling ratio of € 40,000/QALY gained from the societal perspective. Based on the SF-6D utility the mean incremental cost-effectiveness ratio were inferior from both the health care as the societal perspective.

**Table 5:** Incremental costs, effects, and cost-effectiveness analysis

*Base case*	*Adults*					*Children*				
Incremental costs										

Health care costs	*€508*					*€605*				

(2.5^th^–97.5^th ^percentile)	*(-114 to 1,118)*					*(319 to 862)*				
Societal costs	*€1,026*					*€609*				
(2.5^th^–97.5^th ^percentile)	*(231 to 1,889)*					*(312 to 864)*				

Incremental utility adjusted for baseline differences by multiple regression										

EQ-5D QALY^1^	*0.03*					*0.01*				

(2.5^th^–97.5^th ^percentile)	*(0.00 to 0.07)*					*(0.00 to 0.02)*				
SF-6D QALY^1^	*-0.01*									
(2.5^th^–97.5^th ^percentile)	*(-0.07 to 0.03)*					-				

Incremental cost effectiveness ratio										

Health care perspective EQ-5D	*€15,366/QALY gained*					*€58,726/QALY gained*				
Distributions on CEA plane	*origin*	*NE*	*NW*	*SW*	*SE*	*origin*	*NE*	*NW*	*SW*	*SE*
	*0%*	*91%*	*3%*	*0%*	*5%*	*0%*	*98%*	*2%*	*0%*	*0%*
Health care perspective SF-6D	*inferior*					-				
Distributions on CEA plane	*origin*	*NE*	*NW*	*SW*	*SE*					
	*0%*	*17%*	*77%*	*5%*	*1%*					
Societal perspective EQ-5D	*€31,035/QALY gained*					*€59,071/QALY gained*				
Distributions on CEA plane	*origin*	*NE*	*NW*	*SW*	*SE*	*origin*	*NE*	*NW*	*SW*	*SE*
	*0%*	*96%*	*4%*	*0%*	*1%*	*0%*	*98%*	*2%*	*0%*	*0%*
Societal perspective SF-6D	*inferior*					-				
Distributions on CEA plane	*origin*	*NE*	*NW*	*SW*	*SE*					
	*0%*	*17%*	*83%*	*0%*	*0%*					

*Sensitivity analysis (minus asthma monitor costs)*										

Incremental costs										

Health care costs	*€58*					*€156*				

(2.5^th^–97.5^th ^percentile)	*(-544 to 671)*					*(-137 to 411)*				
Societal costs	*€576*					*€159*				
(2.5^th^–97.5^th ^percentile)	*(-257 to 1,423)*					*(-142 to 417)*				

Incremental cost effectiveness ratio										

Health care perspective EQ-5D	*€1,759/QALY gained*					*€15,092/QALY gained*				
Distributions on CEA plane	*origin*	*NE*	*NW*	*SW*	*SE*	*origin*	*NE*	*NW*	*SW*	*SE*
	*0%*	*91%*	*3%*	*0%*	*5%*	*0%*	*84%*	*1%*	*0%*	*15%*
Health care perspective SF-6D	*inferior*					-				
Distributions on CEA plane	*origin*	*NE*	*NW*	*SW*	*SE*					
	*0%*	*9%*	*46%*	*38%*	*7%*					
Societal perspective EQ-5D	*€17,427/QALY gained*					*€15,438/QALY gained*				
Distributions on CEA plane	*origin*	*NE*	*NW*	*SW*	*SE*	*origin*	*NE*	*NW*	*SW*	*SE*
	*0%*	*89%*	*3%*	*0%*	*8%*	*0%*	*85%*	*2%*	*0%*	*13%*
Societal perspective SF-6D	*inferior*					-				
Distributions on CEA plane	*origin*	*NE*	*NW*	*SW*	*SE*					
	*0%*	*14%*	*76%*	*8%*	*2%*					

*Sensitivity analysis (school costs included in analysis)*										

Incremental costs										

Societal costs	*€542*									
(2.5^th^–97.5^th ^percentile)	*(196 to 849)*									

Incremental cost effectiveness ratio										

Societal perspective EQ-5D	*€52,618/QALY gained*									
Distributions on CEA plane						*origin*	*NE*	*NW*	*SW*	*SE*
						*0%*	*97%*	*3%*	*0%*	*0%*

Costs year 2002 in euro.

ICER = Incremental Cost-effectiveness Ratio.

Percentiles based on bootstrap procedure with 5000 replicates.

^1 ^Utility scores at follow-up measurements are adjusted for between group baseline difference using the multiple regression method.

Among the children health care costs were € 605 higher in the intervention group (2.5^th ^percentile € 319; 97^th ^percentile € 862). The difference in societal costs was about the same (€ 609; 2.5^th ^percentile € 312; 97^th ^percentile € 864). The utility adjusted for baseline differences by multiple regression on average 0.01 QALY (2.5^th ^percentile 0.00; 97^th ^percentile 0.02) was gained from the intervention. From the health care perspective the mean ICER was € 58,726/QALY and from the societal perspective € 59,071/QALY gained. The cost-effectiveness acceptability curve show that the probability of acceptance is 68% at a ceiling ratio of € 80,000/QALY gained and 22% at a ceiling ratio of € 40,000/QALY gained from the societal perspective.

### Sensitivity analysis

Table 5 presents a sensitivity analysis in which the monitor costs were set to zero and a sensitivity analysis in which the costs resulting from school absenteeism were included.

**Figure 1:**
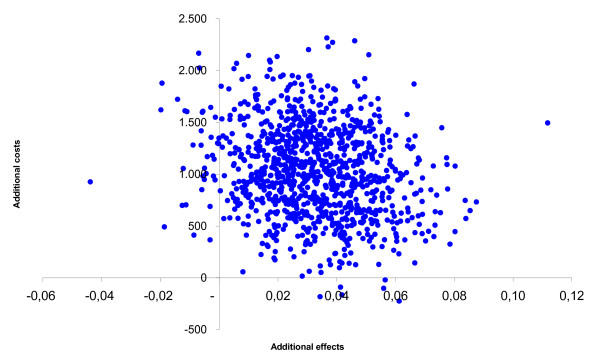
Cost-effectiveness plane from a societal perspective with EQ-5D utility for adults.

**Figure 2:**
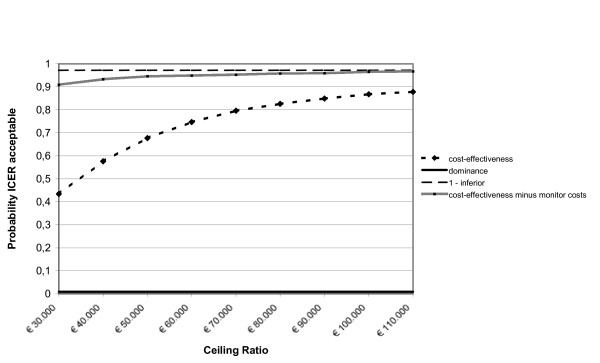
Cost-effectiveness acceptability curves (base case and minus the asthma monitor costs) from a societal perspective with EQ-5D utility for adults.

**Figure 3:**
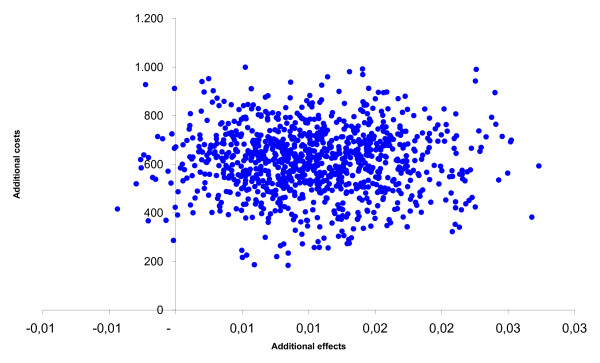
Cost-effectiveness plane from a societal perspective with EQ-5D utility for children.

**Figure 4:**
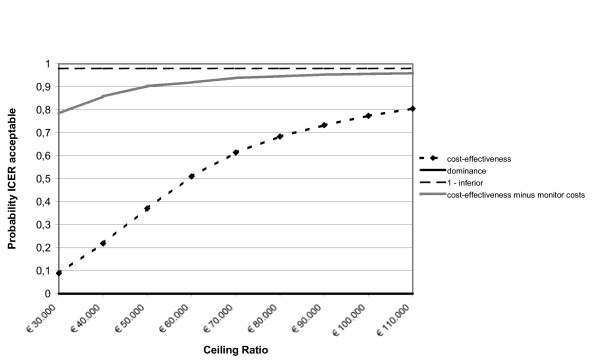
Cost-effectiveness acceptability curves (base case and minus the asthma monitor costs) from a societal perspective with EQ-5D utility for children.

**Figure 5:**
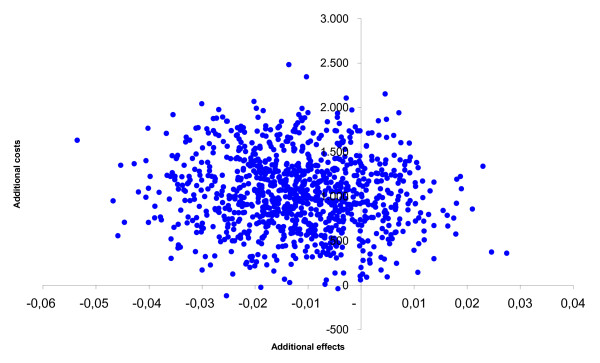
Cost-effectiveness plane from a societal perspective with SF-6D utility for adults.

**Figure 6:**
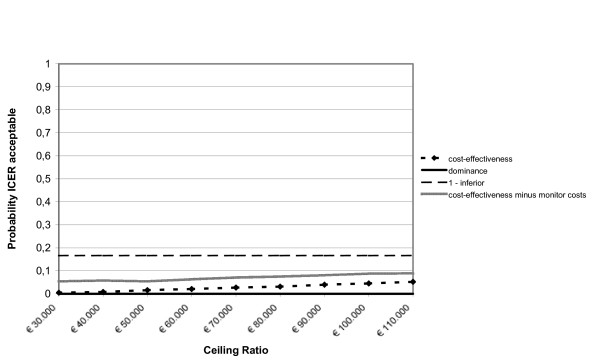
Cost-effectiveness acceptability curves (base case and minus the asthma monitor costs) from a societal perspective with SF-6D utility for adults.

After leaving out the monitor costs, among the adults the incremental health care costs changed from € 508 to € 58. The incremental societal costs decreased from € 1,026 to € 576. From the health care prospective the mean incremental cost-effectiveness ratio changed from € 15,366/QALY gained to € 1,759/QALY gained and from the societal perspective from € 31,035/QALY gained to € 17,427/QALY gained. The probability of cost-effectiveness at a ceiling ratio of € 80,000/QALY gained, changed from 85% to 90% from the societal perspective. The incremental cost-effectiveness ratios based on the SF-6D utility remained inferior. Among the children the incremental health care costs changed from € 605 to € 156 and the societal costs from € 609 to € 159. From the societal perspective the cost-effectiveness ratio changed from € 59,071/QALY gained to € 15,438/QALY gained. The probability that that the intervention was cost-effective changed from 68% to 93% at a ceiling ratio of € 80,000/QALY gained. The figures 1, 2, 3, 4, 5 and 6 present cost-effectiveness curves and cost-effectiveness planes of the base case and the sensitivity analyses, in which the monitor costs were subtracted.

In the control group 7 children had on average 22 hours of school absenteeism and in the intervention group 6 children had on average 5 hours of school absenteeism. As a result the costs of school absenteeism were € 86 (SD € 272) in the control group and € 20 (SD € 85) in the intervention group. Taking these costs into account the incremental societal costs changed from € 609 to € 542 (2.5^th ^percentile € 196; 97^th ^percentile € 849). The cost-effectiveness ratio changed from € 59,071/QALY gained to € 52,618/QALY gained. At a ceiling ratio of € 80,000/QALY gained, the probability of cost-effectiveness changed from 68% to 73% from the societal perspective.

## Discussion

This study compared the cost-effectiveness of a nurse-led telemonitoring programme in asthmatic outpatients to regular care. Patients were asked to transfer their monitor data at least twice daily and by judging the received data and following a stepwise intervention protocol the nurse practitioner was able to act as the main caregiver in the intervention group. In both groups the EQ-5D and the SF-6D were used to obtain estimates of health state utilities. One year, health care costs, patient and family costs, productivity losses were calculated. The mean incremental costs were weighted against the mean incremental effects in terms of QALY.

No significant differences were found between the groups with regard to generic quality of life. Overall, the mean health care costs per patient were higher in the intervention group than in the control group. The intervention costs mainly caused the cost difference between the groups, but not totally. The intervention costs the society € 31,035/QALY gained among adults and among children € 59,071/QALY gained. From the societal perspective the probability of cost-effectiveness is 85% at a ceiling ratio of € 80,000/QALY gained among the adults and 68% among the children. When the QALY was based on the SF-6D utility the mean incremental cost-effectiveness ratios were inferior from both the health care as the societal perspective. In conclusion the intervention appears to be more cost-effective among adults.

If the monitor costs are excluded the incremental costs decrease strongly among both the adults and the children. The decrease of monitor costs results in a far more cost-effective intervention. Given the fast developments in information communication technology, this decrease in price is expected to take place in the future. If the costs with regard to the children's absence from school are included, the cost-effectiveness ratio only decreases about € 6,000, and the probability of cost-effectiveness is still moderate. With regard to the higher costs due to productivity losses in the intervention group caution should be taken, as in the intervention group a greater proportion of patients were paid employers.

Considering the results of this study the intervention is certainly not cost saving, and especially among children, of limited cost-effectiveness. This result is in contrast with the results of a recent review, in which was found that only in three out of twenty-one economic evaluations self-management programmes based on peak flow monitoring, the total costs were higher in the intervention group [25].

As described in a review of Gibson et al. (2003), education in asthma-management which involves self-monitoring, regular medical review, and a written action plan appear to improve health outcomes the most. In this study no written action plans were used, which may have reduced the effectiveness. Therefore, it might be recommended to entail this aspect in future asthma self-management programmes. Furthermore, it is necessary to be cautious in generalizing these findings, as this is an one centre study in a Dutch outpatient setting. Also, these results should not be generalized to asthmatics with more severe symptoms or the results of economic evaluations with a longer time horizon than one year. Another study limitation is the use of interpolated diary data, which can lead to an underestimation or overestimation of the actual resource use during the follow-up year. However, administrating diaries daily during one year is very aggravating and may negatively influence recruitment and drop out. In general, diaries are an accepted way to measure resource use and productivity losses [5,18]. Moreover, any bias is likely to have occurred in both groups. Furthermore, the questionnaire and diary compliance were very high. For these reasons a simple strategy to impute missing data seems justified.

In general the study population had mild to moderate asthma and the patients did not report many asthma symptoms. This is in accordance with the observed high scores in the self-reported quality of life. Nevertheless these patients were diagnosed with asthma and received regular outpatient care for that reason. Home monitoring has been suggested to be the most efficient for asthmatics who are poor perceivers of asthma severity (Fishwick 1996). On the other hand, asthma patients with moderate till severe asthma may have more benefit of the intervention, since there is more room for improvement. This is confirmed in the review of Liljas et al. (1994), in which was stated that asthma self-management seems to be even more cost-effective in patients with severe asthma. The low variation in the generic quality of life scores may also imply that the questionnaires were not sensitive enough for this particular group, especially among children. Though the patients only reported mild asthma symptoms, the health care costs in the study population were relatively high, mainly caused by the intervention and medication costs. This stresses the importance of health services research in asthma self-management programmes, in order to maximise the effects and minimise the costs of medical therapy.

Another reason why the intervention was of limited cost-effectiveness could be that the intervention was initiated separately instead of integrated into the total process of care. That way the intervention may only partially contribute to the improvement of the total care process, both in terms of health outcomes and costs. This underlines the importance of integrated care in telemonitoring programmes.

## Conclusion

In conclusion, if the outcome is measured by generic quality of life the nurse-led telemonitoring programme is of limited cost-effectiveness in the study population. From the societal perspective the probability of the programme being cost-effective compared to regular care was 85% at a ceiling ratio of € 80,000/QALY gained among the adults and 68% among the children. However, a decrease in the price of the asthma monitor will substantially increase the probability of the programme to be cost-effective.

## List of abbreviations used

ICER: incremental cost-effectiveness ratio

QALY: quality adjusted life years

SD: standard deviation

## Competing interests

The author(s) declare that they have no competing interests.

## Authors' contributions

All authors participated in designing the study. DW drafted the manuscript and performed the statistical analyses and MJ contributed to the acquisition. All authors have critically revised the manuscript for scientific content, attributed to the interpretation of the data and approved the final version.
